# Lifestyle Patterns Begin in Early Childhood, Persist and Are Socioeconomically Patterned, Confirming the Importance of Early Life Interventions

**DOI:** 10.3390/nu12030724

**Published:** 2020-03-09

**Authors:** Sandrine Lioret, Karen J. Campbell, Sarah A. McNaughton, Adrian J. Cameron, Jo Salmon, Gavin Abbott, Kylie D. Hesketh

**Affiliations:** 1 Research Center in Epidemiology and Biostatistics (CRESS), Université de Paris, INSERM, INRA, 75004 Paris, France; 2 Institute for Physical Activity and Nutrition (IPAN), School of Exercise and Nutrition Sciences, Deakin University, Geelong, VIC 3220, Australia; karen.campbell@deakin.edu.au (K.J.C.); jo.salmon@deakin.edu.au (J.S.); gavin.abbott@deakin.edu.au (G.A.); kylie.hesketh@deakin.edu.au (K.D.H.); 3 Global Obesity Centre (GLOBE), Institute for Health Transformation, School of Health and Social Development, Deakin University, Geelong, VIC 3220, Australia; adrian.cameron@deakin.edu.au

**Keywords:** diet, physical activity, sedentary behaviour, energy balance-related behaviours, lifestyle patterns, early childhood, tracking

## Abstract

Traditional approaches to understanding the behavioural determinants of adiposity have considered diet, physical activity and sedentary behaviour in isolation. Although integrative approaches have identified a variety of lifestyle patterns in children at preschool-age or older, along with some variability by socio-economic positions, this has rarely been examined in younger cohorts. We aimed to identify lifestyle patterns at 1.5, 3.5 and 5 years, including dietary intake, outdoor time and television viewing time, to assess associations with maternal education (as a proxy for socio-economic position), and to investigate their persistence between toddlerhood and preschool age. Participants were 417 and 293 children aged 1.5 y from the Melbourne Infant Feeding Activity and Nutrition Trial (InFANT) and InFANT Extend Programs, respectively. Data were collected using questionnaires at child ages 1.5, 3.5 and 5 y (InFANT); and 1.5 and 3.5 y (InFANT Extend). Principal component analysis was undertaken at each time point on the separate and pooled datasets. Associations between the lifestyle patterns scores and maternal education were assessed with multivariable regression analysis. Two lifestyle patterns (“Discretionary consumption and TV” and “Fruit, vegetables and outdoor”) were identified as early as 1.5 y. They remained consistent across ages and were evident in both datasets. These patterns were inversely and positively associated with maternal education, respectively. Such early clustering of obesity related energy balance behaviours and tracking during early childhood suggests there may be shared antecedents common to the individual behaviours that could be targeted for intervention. Our findings provide support for interventions targeting multiple behaviours and tailored to the level of family socio-economic disadvantage.

## 1. Introduction

Childhood overweight and obesity is common from as early as two years of age and increases the probability of persistent unhealthy weight into adulthood [1,2], impairing both physical and mental health across the life-course [3]. Beyond genetic or epigenetic susceptibilities, unhealthy lifestyle behaviours such as energy-dense, micronutrient-poor dietary intake, and high levels of screen time (mainly television viewing) are known to promote overweight and obesity [4,5]. In contrast, a healthier diet and physical activity are known to be protective [5,6].

Traditional approaches to understanding the determinants of adiposity have considered these lifestyle behaviours in isolation; however this approach fails to account for the known correlation or clustering of such behaviours in individuals [7]. Applying more integrative approaches based on pattern analyses, a number of studies conducted in children of preschool-age or older have identified a variety of patterns combining both healthy and unhealthy behaviours [8,9]. The two most commonly observed lifestyle patterns in children are a “Snacking and sedentary” pattern, characterized by high consumption of energy-dense or processed foods along with high screen time (mostly television); and a “Healthy diet and physically active” pattern, often characterized by high consumption of core foods such as fruit and vegetables, along with high levels of physical activity. These two socio-economically differentiated lifestyle patterns are important as they reflect inter-related behavioural combinations that contribute either positively or negatively to energy balance, and have been shown to be associated with higher vs. lower risk of obesity in children [8,9,10,11,12]. Analysing the socio-demographic characteristics of the population groups exposed to co-occurrence or clustering of unhealthy behaviours can also inform the targeting of obesity prevention interventions.

While there is evidence to suggest that dietary habits, physical activity and sedentary screen behaviour are established early and remain constant across childhood [13,14,15] and into adulthood [16], to our knowledge, only one study (in two-year-olds) has been published on the clustering of lifestyle behaviours prior to preschool age [17]. No study has assessed whether lifestyle patterns appear earlier than two years, nor their persistence throughout early childhood.

To address this gap, the objectives of the present study were to identify lifestyle patterns at 1.5, 3.5 and 5 years, including dietary intake, outdoor time and television viewing time, to assess their associations with maternal education (used as a proxy for socio-economic position), and to investigate the extent to which these patterns are stable from toddlerhood to preschool age.

## 2. Materials and Methods

### 2.1. Study Design and Participants

The Melbourne Infant Feeding Activity and Nutrition Trial (InFANT) and InFANT Extend Programs are separate cluster randomized controlled trials, which have been described in detail elsewhere [18,19,20]. For both interventions, eligibility involved being a first-time mother of an infant aged around 4 months of age and being able to communicate in English. Of those eligible, 86% (n = 542) and 97% (n = 514) consented to participate in the InFANT and InFANT Extend Programs, respectively. Given the similarity of studies and measures, we pooled data in order to increase power and therefore robustness of the current analyses.

The InFANT Program was a two arm cluster randomised controlled trial that commenced in 2008. Eligible mothers and their infants were recruited from 62 first-time parent groups organized by the universal Maternal and Child Health service provided across Victoria, Australia [18,19]. Families were randomised at the group level to the control or intervention condition. Parents in the intervention group were offered six 2-h dietitian delivered sessions over 15 months, focusing on parental knowledge, skills, and social support around infant feeding, diet, physical activity, and television viewing. The primary aim was to improve lifestyle behaviours and BMI z-scores. Measures were collected at baseline (mean age 4 months), end of intervention (mean age 20 months), and at follow-ups 2 and 3.5 years post intervention (child age approx. 3.5 and 5 y, respectively). Ethical approval was granted by Deakin University’s Human Ethics Research Committee (EC175-2007) and the Victorian Office for Children (CDF/07/1138) and the trial was registered with the ISRCTN Register (ISRCTN81847050).

The Extended Infant Feeding, Activity and Nutrition Trial (InFANT Extend) employed similar methodology to the Melbourne InFANT Program [20]. This trial tested the efficacy of an extended (33 vs. 15 month) and enhanced (use of web-based materials, and Facebook^®^ engagement), version of the original Melbourne InFANT Program intervention in a new cohort of 514 families from 62 first-time parent groups in Melbourne, Australia, from 2010. Measures were collected at baseline (mean age 4 months), mid-intervention (mean age 19 months), and at intervention conclusion (mean age 38 months). Ethical approval was granted by Deakin (EC-175-2007 (Part 2- 2007-175) and the Department of Education and Early Childhood Development (Victoria, Australia) (2011_001000) and was registered with ACTRN (ACTRN12611000386932).

### 2.2. Measurements

Data used in the current study were collected using self-administered questionnaires provided to the main carer (mostly mothers). Eight variables describing usual dietary intake, outdoor time and television time, available at all relevant time points across both studies, i.e., 1.7, 3.5 and 5 y in InFANT and 1.6 and 3.2 y in InFANT Extend, were included in the pattern analyses. For the sake of simplicity, we refer to these three ages as 1.5 y, 3.5 y and 5 y throughout the manuscript, although due to scheduling of visits not all children were the exact age at measurement.

#### 2.2.1. Dietary Intake

Usual dietary intake over the last month was assessed using a 66-item food frequency questionnaire (FFQ) (under review). Nine possible response categories ranged from ‘never or less than once a month’ to ‘6 or more times a day’. Portion sizes for each FFQ item were generated based on median portion sizes consumed at each age in the 2007 National Children’s Nutrition and Physical Activity Survey data [21] and applied to each FFQ item. Daily consumption of each FFQ food item in grams was calculated by converting the frequency of consumption into daily equivalents (Never = 0; 1–3/month = 0.067; 1/week = 0.143; 2–4/week = 0.429; 5–6/week = 0.786; 1/day = 1.0; 2–3/day = 2.5; 4–5/day = 4.5; ≥6/day = 6.0 times per day) and then multiplying by the calculated median portion size for that food to generate grams per day (g/d). Consistent with the main dietary outcomes of the InFANT [18,19] and InFANT Extend [20] protocols, six food/drink groups were retained for inclusion in the current lifestyle pattern analysis, i.e., fruit, vegetables, water, sweet drinks, discretionary sweet foods, and discretionary savory foods (all measured in g/d). There was a ceiling effect for water intake, as about half of parents reported their child consumed six or more serves/d (converted to 1050 g/d). This variable was therefore dichotomized as consuming ≥1050 g/d of water vs. <1050 g/d at each of the time points. Likewise, sweet drinks were consumed by <50% of the children at 1.5 y in both InFANT and InFANT Extend, and were dichotomized into consumers vs. non-consumers at this age. Because we used a pattern analysis sensitive to outliers (see Statistical Analyses section), values for outliers were replaced by the maximum values of the remaining distributions at each age in the InFANT and InFANT Extend samples (*n* = 17 and 12 respectively).

#### 2.2.2. Outdoor Time

Parents reported the number of minutes (open response) their child spent outside on a typical day. Parent-reported usual outdoor time has been shown to be associated with direct objective measurement of physical activity by accelerometer in preschool-aged children [22]. Values for outliers were replaced by the maximum values of the remaining distributions at each age in InFANT and InFANT Extend samples (*n* = 2 and 3 respectively).

#### 2.2.3. Television Viewing Time

Parents reported the number of minutes (open response) their child spent watching or in front of the television on a typical day using items with established reliability [23]. Test-retest reliability in this sample indicated good convergence with intraclass correlation coefficient ICC = 0.69 at 3 months of age and ICC > 0.79 for other ages. Values for outliers were replaced by the maximum value of the remaining distributions at each age in InFANT and InFANT Extend (*n* = 2 and 3 respectively).

#### 2.2.4. Demographic, Socioeconomic and Anthropometric Factors

Demographic, socioeconomic and anthropometric data reported at baseline included children’s sex and birth weight and mother’s country of birth and maternal education level. The latter was defined in three categories: low (secondary school or below), intermediate (trade and certification qualifications) and high (university degree). Maternal education is the indicator most commonly used as a proxy for socio-economic position when considering lifestyle behaviours of children [24]; also, it is consistently and strongly associated with child adiposity in the literature [25] and is less likely to be affected by motherhood, as compared to other indicators such as income and occupation.

#### 2.2.5. Included Samples

**Figure 1:**
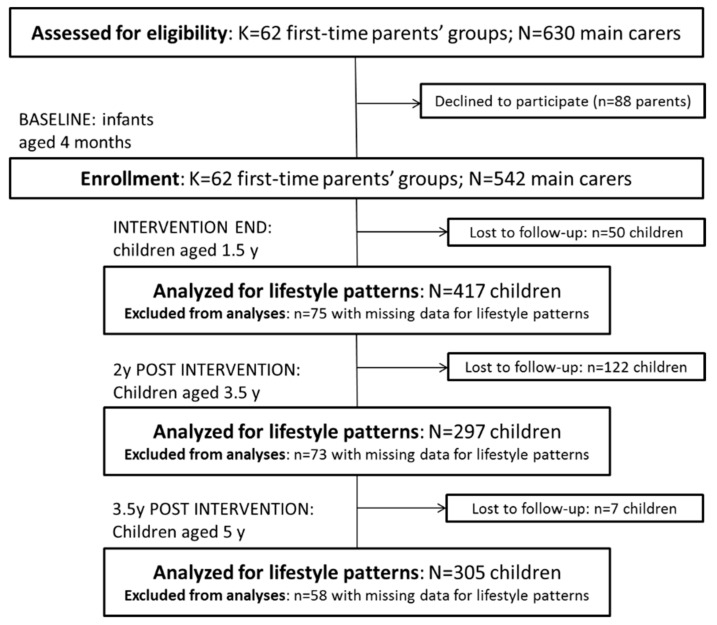
Flow of participants through the Melbourne InFANT Program.

**Figure 2:**
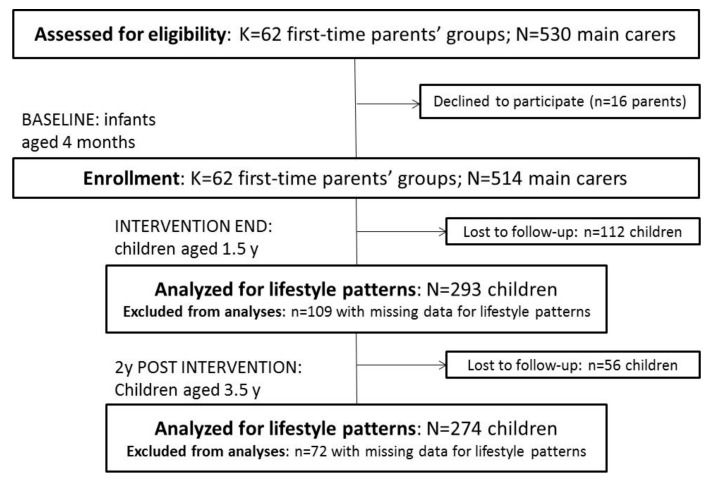
Flow of participants through the Melbourne InFANT Extend Program.

Of the 542 and 514 mother–child dyads initially recruited to InFANT and InFANT Extend, respectively, 179 and 168 participants dropped out between enrolment and the end of follow-up, i.e., at 5 y in InFANT and at 3.5 y in InFANT Extend ( Figure 1; Figure 2). Additionally, children with missing information on any of the behaviours of interest for the current study were excluded from the analysis (*n* = 75 at 1.5 y; *n* = 73 at 3.5 y; and *n* = 58 at 5 y for InFANT; *n* = 109 at 1.5 y; and *n* = 72 at 3.5 y for InFANT Extend), resulting in 417, 297 and 305 eligible children for analysis at 1.5, 3.5 and 5 y in InFANT; and 293 and 274 at 1.5 and 3.5 y in InFANT Extend.

We derived lifestyle patterns for all subjects with complete data at each age. Multi-time-point lifestyle patterns were also assessed in the 247 and 210 children with data available at all three ages in InFANT and at the two ages in InFANT Extend, respectively.

### 2.3. Statistical Analyses

We described the children from the InFANT (*n* = 417) and InFANT Extend (*n* = 293) datasets who had all behavioural data available at approx. 1.5 y. Socio-demographic and anthropometric factors were then compared between this sample and the children assessed at baseline (children aged 4 months) but not included in the analysis undertaken at 1.5 y, because of loss to follow-up or missing data (*n* = 125 and *n* = 221, respectively). Chi-square and Student *t* tests were used to compare frequencies and means, respectively.

Different data-driven approaches have been used to identify both dietary and lifestyle patterns, in particular principal component analysis (PCA) and cluster analysis (CA). These approaches have shown good reproducibility [8,9,26]. PCA allows the synthesis of a large number of supposedly correlated variables into a fewer number of patterns, which are linear combinations of the initial standardized variables: each subject from the analytical sample has a score on each of the patterns derived. CA reduces data into mutually exclusive groups (clusters) based upon individual differences in mean intakes: subjects are member of one given cluster. In the current analysis, we aimed to measure adherence to each of the patterns derived, and not separate children in mutually exclusive groups, which led us to use PCA.

Cross-sectional lifestyle patterns were first derived independently at 1.5 y and 3.5 y (InFANT and InFANT Extend) and 5 y (InFANT) using PCA with varimax rotation of the 8 standardized behavioural variables, i.e., fruit, vegetables, water, sweet drinks, discretionary sweet foods, discretionary savory foods, outdoor and television viewing time. The number of patterns was selected considering eigenvalues >1.0, the scree plot and the interpretability of the patterns [27,28]. To interpret and label each pattern, we considered the items most strongly related to that pattern, i.e., those for which the absolute value of the loading coefficient (i.e., the correlation of each variable with the given lifestyle pattern) was >0.25. The PCA scores for each lifestyle pattern were calculated at the individual level by summing the observed standardized values of each variable, weighted according to the PCA loadings.

Longitudinal, multi-time-point lifestyle patterns were then derived in a separate PCA with varimax rotation, for each study, including simultaneously the standardized variables defined at all available time points, i.e., 1.5 y, 3 y and 5 y (InFANT, n = 247); and 1.5 y and 3 y (InFANT Extend, *n* = 210). The same criteria were considered to retain and label the resulting patterns. This longitudinal application of PCA was previously used by Brazionis et al. [29] to assess transition diets in the early years and by Lioret et al. [14] as a longitudinal measure of tracking on dietary patterns. Persistence of patterns from 1.5 y to 5 y (InFANT, *n* = 247) and from 1.5 y to 3.5 y (InFANT Extend, *n* = 210) was also assessed with Pearson correlation coefficients and associated *p*-values estimated between lifestyle patterns in each dataset. Recommendations for interpreting these correlation coefficients are: low 0.1–0.3, medium 0.3–0.5 and high >0.5 [30]. Sensitivity pattern analyses were undertaken on the pooled InFANT and InFANT Extend datasets at 1.5 y (*n* = 710) and 3.5 y (*n* = 571). Of note, consistent loading coefficients were observed from PCA independently undertaken in intervention and control groups (results available on request); findings from the whole InFANT and InFANT Extend samples were, however, prioritized to allow a higher statistical power and therefore precision of the estimated scores. Lastly, we performed multivariable linear regression analysis to investigate the relations between a given lifestyle pattern (as the outcome) and maternal education level, adjusting for child sex, age, treatment and using the survey command in Stata (to account for clustering by first-time parent group). This analysis was performed in each of the pooled 1.5 y and 3.5 y samples to avoid multiple comparisons. Adjusted parameter estimates and 95% CIs were calculated.

## 3. Results

### 3.1. Characteristics of the Study Population

Children were aged on average 1.6 (SD = 0.2), 3.6 (0.2), and 5.0 (0.1) y at the three time points in InFANT; and 1.6 (0.1) and 3.2 (0.1) y at the two time points in InFANT Extend. Sample characteristics are presented in Table 1. In InFANT Extend, the 221 children excluded from the analyses at 1.5 y due to loss at follow-up or missing data came from families where the mothers were younger and less likely to have achieved a high education level compared to those retained in the analyses; there were no such differences in InFANT.

### 3.2. Characteristics of Lifestyle Patterns

#### Cross-Sectional PCA Approach

Two consistent lifestyle patterns were identified in each of the independent analyses across time points and study populations. Each of these patterns accounted for 18.3–22.0% of the explained variance (Table 2). Overall, at each time point, one pattern was consistently and positively correlated with intakes of sweet drinks, discretionary sweet foods, discretionary savory foods and television time. We labelled this pattern “Discretionary consumption and TV” (referred to as LP1 in the tables). The other pattern, labelled “Fruit, vegetables and outdoor” (referred to as LP2 in the tables), was mainly characterized by high intake of fruit and vegetables and high levels of outdoor time. When the absolute value of water intake loaded 0.25, water was either negatively correlated with the “Discretionary consumption and TV” pattern, or positively correlated with the “Fruit, vegetables and outdoor” pattern. Consistent findings were obtained from analyses using the pooled InFANT-InFANT Extend dataset at 1.5 and 3.5 y (Table 3).

### 3.3. Persistence of Lifestyle Patterns from Toddlerhood to Preschool Age

#### 3.3.1. Longitudinal PCA Approach

Consistent with the findings in the cross-sectional approach, in longitudinal analyses, we identified the two multi-time-point lifestyle patterns, “Discretionary consumption and TV” and “Fruit, vegetables and outdoor”, spanning 1.5 to 5 y in InFANT and 1.5 to 3.5 y in InFANT Extend. These accounted for 13.2–16.4% of the explained variance (Supplementary Materials Table S1).

#### 3.3.2. Correlation Approach

Correlation coefficients between the “Discretionary consumption and TV” pattern observed at 1.5 y, 3.5 y and 5 y respectively were universally high (0.50–0.63) [30], with corresponding coefficients for the “Fruit, vegetables and outdoor” pattern at different ages being medium to high (0.36–0.60) (Table 4).

### 3.4. Associations with Maternal Education

The “Discretionary consumption and TV” pattern was inversely associated with maternal education level at both 1.5 (significant trend) and 3.5 y in the pooled datasets (Table 5). Conversely, the “Fruit, vegetables and outdoor” pattern was positively associated with maternal education at 1.5 y.

## 4. Discussion

To the best of our knowledge, this study is the first to show that lifestyle patterns, namely “Discretionary consumption and TV” and “Fruit, vegetables and outdoor”, are established by 1.5 years of age. Further, there is evidence of persistence of these lifestyle patterns across early childhood, and that these patterns are influenced by maternal education.

Similar patterns to those identified in this study have been reported in other studies of early childhood populations. In the study most comparable to ours, using PCA, Gubbels et al. [17] found a “Snacking-sedentary” pattern in toddlers aged 2 y in the Netherlands, which was characterized by eating snacks, drinking sugar-sweetened beverages, television viewing and computer use. Lifestyle patterns characterized by less healthy foods and drinks, and screen time, have also been described using either PCA or cluster analysis in children aged 3–6 years across Europe [31,32,33]. This consistency across countries, datasets and methods used to derive such patterns has also been confirmed in school-aged children [8,9,10,34,35,36]. Consistent with our findings, these lifestyle patterns were typically inversely associated with parental socio-economic positions in other studies, most commonly approximated by maternal education [8,9,35]. The co-occurrence between television viewing and unhealthy dietary habits has often been described in the literature. Proposed mechanisms include: watching TV or movies as a hand-free passive occupation enables snacking; exposure to advertising of junk food and fast food when watching TV may increase children’s requests for those particular foods and products; and viewing screens while eating distracts from internal satiety cues potentially leading to overconsumption [37,38,39]. Notably, a lifestyle pattern characterized by consumption of discretionary foods and drinks, and elevated screen time, is the pattern most often associated with child overweight in the literature [8,9,10,11,12,35,36].

The lifestyle pattern characterized by the high consumption of core foods such as fruit and vegetables, along with increased time spent in physical activities (referred to here as “Fruit, vegetables and outdoor”) has been less frequently identified in other studies, and only in children aged 3 y or older [8,9,10,36]. Among pre-schoolers, Lioret et al. [31] identified a “Varied food and physically active” pattern in 3–6 y French children; and Miguel-Berges et al. [33] identified a “Healthy lifestyle” pattern in European preschool-aged children (3.5–5.5 y), characterized not only by high water, fruit, vegetable intake and sports participation, but also by increased sleep time; and reduced soft drinks and screen time. A very similar healthy pattern was described in Australian children aged 6–7 y [10]. This lifestyle pattern, combining a range of healthy behaviours, has most often been found to be positively associated with parental socio-economic position (often approximated by maternal education) [8,9,10], which our findings confirm at 1.5 y. This socioeconomic disparity may reflect varying family knowledge, social norms, attitudes, and lifestyles, within an environment providing differential access to both healthy food and safe places to play outside. It is important to note that young children are mostly controlled and influenced by their parents, who are also usually their main role models and providers [40]. There is some evidence to suggest that children’s lifestyle, whether diet, physical activity or sedentary behaviours, is strongly associated with their parents’ diet and physical activity, and with parent capacity to control the availability of healthy foods at home and engage their children in physical activities [41,42,43]. Potentially, the same parents who are aware of, value, and have the resources to provide a healthy diet, will also be aware of, value and have the resources to promote physical activity and limit screen time, leading to an “overall healthy” lifestyle pattern.

Tracking from early life of each of the lifestyle behaviours under study has been previously described [13,14,15]. The present study, however, further highlights that combinations of either healthy or unhealthy behaviours persist from as early as 1.5 y, which provides new and important insights into the genesis of these behaviours. Not only have we shown that similar lifestyle patterns were overall highly correlated between 1.5, 3.5 and 5 y in InFANT, especially for unhealthy behaviours, but we have also derived two longitudinal multi-time point patterns, each characterized by repeated exposures to the same behaviours over time. Higher scores for “Discretionary consumption and TV at 1.5, 3.5 and 5 y” reflect higher tracking for this particular pattern of behaviours, whereas higher scores for the “Fruit, vegetables and outdoor at 1.5, 3.5 and 5 y” pattern may be interpreted as a stable adherence to a healthy lifestyle across early childhood. Consistent findings and interpretations from both InFANT and InFANT Extend datasets suggests robustness of these patterns across samples. Another study examining the clustering of energy balance-related behaviours in a smaller sample of Australian school-aged children (123 aged 5–6 and 87 children aged 10–12 y at baseline) found fair to moderate persistence of lifestyle cluster membership over three years, providing further evidence for the early establishment of clusters of obesogenic lifestyle behaviours [35]. In that study, the clusters of obesity-promoting behaviours were hypothesized to lead to cumulative increases in positive energy balance over the life course.

These findings have important implications for conceptualizing early obesity prevention policy and practice. A better understanding of when, which and in whom behaviours co-occur and start to track collectively over time informs the design and delivery of interventions targeting groups of behaviours rather than individual behaviours. Importantly, these findings will also inform policy that guides provision of early childhood care, ensuring that the large numbers of children in childcare are supported to achieve healthy behavioural patterns. It has also been hypothesized that with behaviours that co-occur, change in one may support change in the other, highlighting the potential for multiple-behaviour interventions to have a greater impact on public health than single-behaviour interventions, especially for preventing multifactorial health conditions [44,45]. Interventions focusing on reducing television time, along with the promotion of physical activity engagement and healthy eating, therefore may be promising, especially in children of less educated mothers. Such integrated interventions may have the potential to provide a synergistic impact on health behaviours that goes beyond the simple additive effects of focusing on individual behaviours consecutively. This may help explain the greater success of multi-component interventions in the prevention of childhood overweight [44,45].

### Limitations and Strengths

In interpreting the findings of the present study, it is important to acknowledge several limitations. Parental self-report of children’s behaviours is subject to measurement error, i.e., social desirability bias and relative imprecision as compared to objective measurements (e.g., using accelerometry for physical activity). With respect to food intake, frequency of consumption has been shown to be the major determinant of dietary intake [46]. The FFQ used across the studies was developed specifically for this age group and has been validated against three 24 h recalls in children aged 1.5, 3.5 and 5.0 y: fair agreement for most nutrient and food intakes was observed, with acceptable rankings of intake suitable for use in PCA (which is based on the underlying correlation matrix) (under review). Additionally, outdoor time has been positively associated with objectively-measured PA in pre-schoolers [22], and both outdoor and television viewing time have been found to be prospectively related to adiposity in early childhood [6,47]. Of note, whereas accelerometry does provide objective information on duration and intensity of activities, the measurements of physical activity and sedentary screen behaviours used in the present study provide information on the context in which they take place (i.e., outdoor and television viewing), which is potentially useful for designing prevention interventions. The present study involved PCA, which is an exploratory statistical alternative among pattern analyses. Strict comparison with other findings is not straightforward due to the data driven approach to pattern analysis, with different methodologies employed in the collection of behavioural data, subjective redistribution of the former items (e.g., foods) into the variables used (e.g., food groups), the transformation of these variables (e.g., standardisation), the methods of rotation of components (if any) employed, and the number of patterns identified—all complicating the opportunities for comparison. Despite these potential variations in methodology, various reviews [8,9,26] have reported fairly good reproducibility between most studies which have identified either dietary or lifestyle patterns. This consistency and therefore robustness was confirmed in the present study. Regarding representativeness, although all levels of maternal education were represented in this study, the sample is generally well educated, with a majority having tertiary education. In addition, mothers excluded from the analyses tended to be less educated and younger, which is a common trait of cohort studies. Whereas these characteristics may have implications for generalizability, the lifestyle patterns derived here are rather consistent with other studies, both cross-sectional and longitudinal, performed in preschool and school-aged children in Australia and overseas.

Particular strengths of the study include consistent measures for a variety of behaviours across studies and time-points, relatively large sample sizes (particularly for pooled analyses), the longitudinal design, and the integrative approach of health behaviours.

## 5. Conclusion

In conclusion, healthy (“Fruit, vegetables and outdoor”) and unhealthy (“Discretionary consumption and TV”) lifestyle patterns were identified in the present study. These patterns were evident as early as 1.5 y of age, and were extremely robust—across samples and at different child ages. Similar patterns have been observed in a number of other studies carried out in older children [7,8,9,29,30,31,32]. Such early clustering and tracking of healthy and unhealthy energy balance-related behaviours suggest there may be shared antecedents common to the individual behaviours that could be targeted for intervention. Finally, the association with maternal education level indicates that such multiple behaviour interventions may be more effective to prevent child overweight if adapted to the level of family social disadvantage.

## Figures and Tables

**Table 1:** Characteristics of the study samples.

	InFANT			InFANT Extend		
	Study Sample at 1.5 y	Children Included at Baseline but Excluded from Analyses at 1.5 y	*p*-Value	Study Sample at 1.5 y	Children Included at Baseline but Excluded from Analyses at 1.5 y	*p*-Value
*n*	417	125		293	221	
Maternal age at baseline, mean (SD)	32.4 (4.2)	32.1 (4.6)	0.54	32.5 (4.3)	31.4 (4.3)	0.008
Maternal education level, *n* (%)			0.21			0.02
Low	85 (20.4)	27 (23.9)		28 (9.6)	31 (16.6)	
Intermediate	98 (23.5)	33 (29.2)		83 (28.5)	61 (32.6)	
High	234 (56.1)	53 (46.9)		180 (61.9)	95 (50.8)	
Mother’s country of birth, *n* (%)			0.26			0.10
Australia	334 (80.1)	85 (75.2)		227 (78.8)	135 (72.2)	
Other	83 (19.9)	28 (24.8)		61 (21.2)	52 (27.8)	
Child sex, *n* (%)			0.17			0.98
Male	226 (54.2)	59 (47.2)		154 (52.6)	100 (52.4)	
Female	191 (45.8)	66 (52.8)		139 (47.4)	91 (47.6)	
Child birth weight, mean (SD)	3376.5 (595.6)	3361.9 (645.1)	0.82	3323.2 (612.1)	3416.2 (554.6)	0.11

**Table 2:** Behavioural variables distribution and PCA loadings for lifestyle patterns (LP) derived at 1.5 y, 3.5 y and 5 y.

Age	1.5 y						3.5 y						5 y		
Study	InFANT			InFANT Extend			InFANT			InFANT Extend			InFANT		
*n*	417			293			297			274			305		
	**Mean (SD) ^a^**	**PCA Loadings**		**Mean (SD) ^a^**	**PCA Loadings**		**Mean (SD) ^a^**	**PCA Loadings**		**Mean (SD) ^a^**	**PCA Loadings**		**Mean (SD)** ^a^	**PCA Loadings**	
Behavioural variables		LP1 ^b^	LP2 ^c^		LP1 ^b^	LP2 ^c^		LP1 ^b^	LP2 ^c^		LP1 ^b^	LP2 ^c^		LP1 ^b^	LP2 ^c^
Fruit, g/d	195.3 (113.0)^d^	0.06	**0.65**	270.0 (140.7)	0.02	**0.59**	248.2 (119.9)	0.02	**0.56**	275.5 (145.2)	0.04	**0.69**	258.9 (129.9)	0.07	**0.54**
Vegetables, g/d	98.5 (50.4)	−0.06	**0.63**	97.3 (50.7)	−0.01	**0.58**	88.8 (50.3)	−0.09	**0.60**	83.8 (47.1)	−0.06	**0.65**	89.4 (46.8)	0.06	**0.52**
Water ≥ 1050 g/d	49.4	−0.17	0.07	57.3	**−0.27**	0.13	38.7	−0.22	**0.29**	47.1	−0.21	0.00	38.0	−0.20	**0.37**
Sweet drinks, g/d	37.6	**0.50**	0.07	34.8	**0.53**	0.02	53.9 (79.9)	**0.44**	−0.05	31.4 (44.4)	**0.50**	0.07	49.9 (71.2)	**0.47**	−0.09
Discretionary sweet foods, g/d	9.6 (10.0)	**0.54**	−0.07	8.3 (9.3)	**0.58**	0.00	15.0 (11.6)	**0.48**	0.00	13.3 (10.3)	**0.47**	0.03	15.2 (12.2)	**0.57**	0.12
Discretionary savory foods, g/d	3.1 (4.1)	**0.55**	0.06	2.9 (3.8)	**0.45**	0.09	4.6 (5.4)	**0.45**	−0.07	5.2 (5.0)	**0.52**	0.01	5.0 (5.2)	**0.53**	−0.08
TV, min/d	51.2 (58.0)	**0.31**	−0.22	47.0 (45.6)	**0.29**	**−0.34**	106.6 (76.8)	**0.51**	0.09	92.5 (59.1)	**0.45**	−0.16	96.9 (64.4)	**0.36**	0.14
Outdoor, min/d	101.7 (69.9)	0.14	**0.33**	123.0 (65.4)	0.18	**0.43**	164.9 (99.2)	0.23	**0.48**	174.9 (105.8)	0.10	**0.26**	160.3 (80.2)	−0.06	**0.50**
% variance explained		18.9	18.5		20.8	20.1		22.0	18.3		21.8	18.4		21.0	19.4

^a^ Values are % yes (water intake; and sweet drinks intake at 1.5 years) or mean (SD); ^b^ LP1: “Discretionary consumption and TV” lifestyle pattern; ^c^ LP2: “Fruit, vegetables and outdoor” lifestyle pattern. ^d^ Fruit was summed consumption of 10 fruits; at 1.5 y in the InFANT sample banana was not reported so fruit was summed consumption of nine fruits. In bold: Factor loadings <−0.25 or >0.25.

**Table 3:** PCA loadings for lifestyle patterns (LP) derived at 1.5 y and 3.5 y in the pooled datasets (InFANT and InFANT Extend studies combined).

Age	1.5 y		3.5 y	
*n*	710		571	
Behavioural variables	LP1	LP2	LP1	LP2
Fruit	0.03	**0.62**	0.04	**0.67**
Vegetables	−0.04	**0.58**	−0.06	**0.64**
Water	−0.21	0.12	**−0.24**	0.15
Sweet drinks	**0.51**	0.07	**0.47**	0.03
Discretionary sweet foods	**0.56**	−0.04	**0.49**	0.02
Discretionary savory foods	**0.51**	0.06	**0.48**	0.02
TV	**0.30**	**−0.27**	**0.46**	−0.07
Outdoor	0.15	**0.43**	0.15	**0.33**
% variance explained	19.6	19.1	21.7	18.0
Label	Discretionary consumption and TV	Fruit, vegetables and outdoor	Discretionary consumption and TV	Fruit, vegetables and outdoor

In bold: Factor loadings <−0.25 or >0.25.

**Table 4:** Pearson correlation coefficients ^1^ and *p*-values between lifestyle patterns (LP) scores identified at 1.5 y, 3.5 y and 5 y.

Study (*n*)	Age		3.5 y		5 y	
			LP1	LP2	LP1	LP2
**InFANT** (247)	1.5 y	LP1	0.52 ***	−0.20 **	0.50 ***	−0.17 **
		LP2	−0.05	0.47 ***	−0.12	0.36 ***
	3.5 y	LP1			0.63 ***	−0.13 *
		LP2			−0.23 ***	0.60 ***
**InFANT Extend** (210)	1.5 y	LP1	0.51 ***	−0.06		
		LP2	−0.04	0.51 ***		

* *p* < 0.05, ** *p* < 0.01, *** *p* < 0.001, *n* = 989; ^1^ Recommendations for interpreting these correlation coefficients: low <0.3, moderate 0.3–0.6 and high >0.6 [30]; LP1: “Discretionary consumption and TV” lifestyle pattern; LP2: “Fruit, vegetables and outdoor” lifestyle pattern.

**Table 5:** Lifestyle pattern scores in the pooled InFANT and InFANT Extend datasets according to maternal education level (Adjusted ^a^ β parameter estimates and 95% confidence intervals).

Lifestyle Pattern	LP1				LP2			
Age	1.5 y		3.5 y		1.5 y		3.5 y	
	β	95% CI	β	95% CI	β	95% CI	β	95% CI
*n*	708		567		708		567	
**Maternal education**								
Low	Ref		Ref		Ref		Ref	
Intermediate	0.01	−0.29; 0.31	−0.34	−0.77; 0.08	0.18	−0.12; 0.49	−0.15	−0.51; 0.20
High	−0.22	−0.50; 0.05	−0.65	−0.98; −0.32	0.34	0.08; 0.59	−0.01	−0.33; 0.32
*p* value	0.06		0.0002		0.03		0.45	
*p* trend	0.037		0.000		0.009			

^a^ Multivariable linear regression analyses accounted for child sex, age, treatment and clustering by first-time parent group. LP1: “Discretionary consumption and TV” lifestyle pattern; LP2: “Fruit, vegetables and outdoor” lifestyle pattern.

## Supplementary Materials

The following are available online at https://www.mdpi.com/2072-6643/12/3/724/s1. Table S1: PCA loadings for the multi-time-point lifestyle patterns (LP) in InFANT from 1.5 y to 5 y and in InFANT Extend from 1.5 y to 3.5 y.
