# Supplementary material for: Lifestyle Patterns Begin in Early Childhood, Persist and Are Socioeconomically Patterned, Confirming the Importance of Early Life Interventions
Source: Nutrients. 2020 Mar 9;12(3):724. doi: 10.3390/nu12030724 (PMC7146362; doi:10.3390/nu12030724)
Supplement: Supplementary file 1 [file nutrients-12-00724-s001.pdf]

Table S1 PCA loadings for the multi-time-point lifestyle patterns (LP) in InFANT from 1.5 y to 5 y and in InFANT Extend from 1.5 y to 3.5 y.

**Study**	**InFANT**						**InFANT Extend**			
n	**247**						**210**			
**Lifestyle pattern**	**LP1**			**LP2**			**LP1**		**LP2**	
Age	1.5 y	3.5 y	5 y	1.5 y	3.5 y	5 y	1.5 y	3.5 y	1.5 y	3.5 y
Behavioural variables										
Fruit [a]	0.03	0.03	0.04	**0.29**	**0.35**	**0.30**	0.03	0.02	**0.45**	**0.44**
Vegetables	0.01	-0.03	0.01	**0.34**	**0.41**	**0.35**	-0.01	-0.02	**0.41**	**0.45**
Water	-0.02	-0.10	-0.10	0.22	0.19	0.20	-0.16	-0.10	0.00	-0.04
Sweet drinks	**0.25**	**0.29**	**0.29**	-0.02	-0.03	-0.03	**0.35**	**0.39**	0.05	0.15
Discretionary sweet foods	**0.29**	**0.33**	**0.32**	0.02	0.05	0.05	**0.36**	**0.33**	-0.07	-0.03
Discretionary savory foods	**0.28**	**0.34**	**0.31**	-0.01	0.02	-0.07	**0.35**	**0.39**	0.02	0.05
TV	**0.26**	0.23	0.16	-0.01	0.05	-0.04	0.24	**0.34**	-0.22	-0.17
Outdoor	0.08	0.05	-0.04	0.15	**0.26**	**0.26**	0.09	0.05	0.22	**0.28**
% variance explained		15.0			13.2		16.4		14.8	
Label	"Discretionary consumption and TV at 1.5, 3.5 and 5 y"			"Fruit, vegetables and outdoor at 1.5, 3.5 and 5 y"			"Discretionary consumption and TV at 1.5 and 3.5 y"		"Fruit, vegetables and outdoor at 1.5 and 3.5 y"	

[a]Fruit was summed consumption of 10 fruits; at 1.5 y in the InFANT sample banana was not reported so fruit was summed consumption of 9 fruits. In bold: Factor loadings <-0.25 or >0.25.
